# Role of the Guanine Nucleotide Exchange Factor GBF1 in the Replication of RNA Viruses

**DOI:** 10.3390/v12060682

**Published:** 2020-06-24

**Authors:** José L. Martínez, Carlos F. Arias

**Affiliations:** Instituto de Biotecnología, Universidad Nacional Autónoma de México, Cuernavaca 4510, Morelos, Mexico; josmtz@ibt.unam.mx

**Keywords:** RNA viruses, GBF1, vesicle transport

## Abstract

The guanine nucleotide exchange factor GBF1 is a well-known factor that can activate different ADP-ribosylation factor (Arf) proteins during the regulation of different cellular vesicular transport processes. In the last decade, it has become increasingly evident that GBF1 can also regulate different steps of the replication cycle of RNA viruses belonging to different virus families. GBF1 has been shown not only to facilitate the intracellular traffic of different viral and cellular elements during infection, but also to modulate the replication of viral RNA, the formation and maturation of viral replication complexes, and the processing of viral proteins through mechanisms that do not depend on its canonical role in intracellular transport. Here, we review the various roles that GBF1 plays during the replication of different RNA viruses.

## 1. Introduction

Eukaryotic cells contain a collection of diverse organelles that perform specialized functions depending on the enzymatic activities of their components. Although these organelles are spatially separated, there is a dynamic interaction between them by different trafficking events, some of which are mediated by vesicle transport [1]. This transport is responsible for the proper intracellular distribution of proteins and lipids and is essential to sustain the correct function and structure of cell organelles [2,3,4,5,6,7]. In vesicle transport, the proteins and lipids to be transported are selectively concentrated on specialized membrane sites of a donor compartment and included into spherical carrier vesicles coated with specific proteins. The vesicles detach from the donor membrane and travel through the cytoplasm until they finally fuse with their target compartment to release their content [8,9,10].

**Figure 1:**
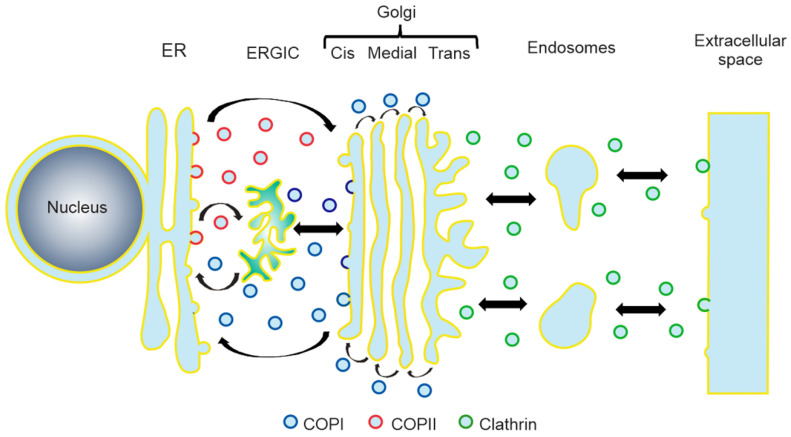
Schematic diagram of the roles of COPI, COPII, and clathrin in the vesicular secretory pathway. ER, endoplasmic reticulum; ERGIC, ER-Golgi intermediate compartment.

Based on the protein elements that form the coat, three important transport vesicles have been extensively described in which the function depends on either the coatomer protein I (COPI), COPII, or clathrin [11,12]. These vesicles mediate different steps of the secretory pathway of the cell. COPII regulates the anterograde transport from the endoplasmic reticulum (ER) to the Golgi apparatus [13,14], whereas COPI facilitates the transport between the Golgi cisternae as well as the retrograde transport from Golgi to the ER [15,16,17]. On the other hand, clathrin-coated vesicles direct the transport from the Golgi to the plasma membrane, as well as between the plasma membrane and endosomes [18] (Figure 1). Recently, besides its canonical functions in the secretory pathway, the COPI machinery has been shown to participate in the maturation of early endosomes and in recycling proteins to the plasma membrane [19,20], as well as in the maturation of phagosomes [21,22] and peroxisomes [23]. Furthermore, multiple reports suggest that COPI-vesicles are also involved in transport events related to the maturation and function of lipid droplets (LDs) [24,25,26].

**Figure 2:**
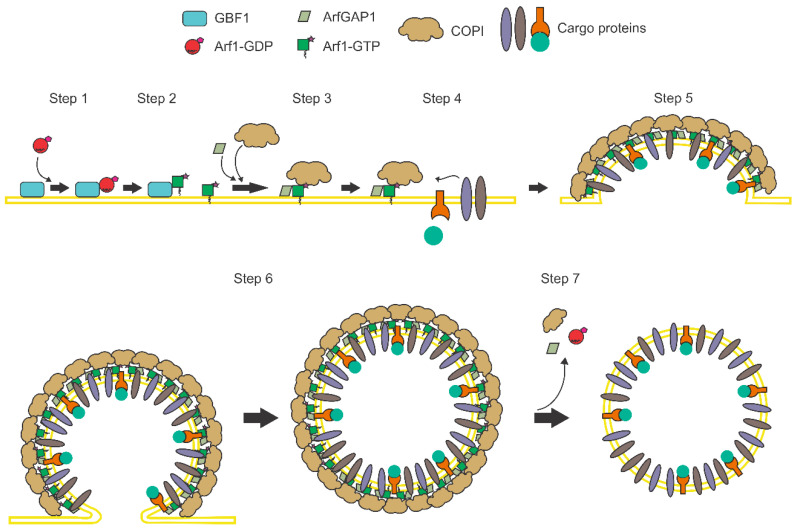
Mechanism of COPI transport. COPI vesicle formation starts when the small GTPase Arf1, bound to GDP (Arf1-GDP), associates with the Golgi-specific Brefeldin A (BFA) resistance factor 1 (GBF1), a guanine nucleotide exchange factor (GEF) that catalyzes the activation of Arf1 by promoting the exchange of GDP for a GTP molecule (step 1). This exchange induces a conformational change in Arf1 that leads to the exposure of a myristoyl group that allows the association of this protein with the membrane [31] (step 2). The membrane-bound Arf1-GTP then promotes the recruitment of the preformed COPI coat formed by seven subunits, called α, β, β’, δ, ε, γ and ζ, and the Arf-GTPase-activating protein 1 (ArfGAP1) (step 3). The formation of the Arf1-GTP-COPI-ArfGAP1 complex in the membranes stimulates the binding and concentration of different cargoes (step 4) and induces the bending of the membrane into a vesicle (step 5). Once the vesicle is completed, it buds from the membrane with a coat of COPI (step 6). Finally, the coat proteins are disassembled when the GTPase activity of Arf1 is enhanced by ArfGAP1, leading to the hydrolysis of the GTP molecule, promoting the release of Arf1-GDP, the COPI subunits, and ArfGAP1 from the vesicle to produce the free carrier vesicle used in the vesicular transport (step 7) [32,33].

The mechanism of COPI vesicle formation is a complex process regulated by the activation of the small GTPase Arf1 (ADP-ribosylation factor 1) (Figure 2). This protein belongs to a family of low molecular weight guanine-nucleotide-binding proteins that can be divided into Arf proteins of Class I (Arf1, Arf2, and Arf3), Class II (Arf4 and Arf5) and Class III (Arf6), depending on their sequence identity. Arf proteins cycle between an inactivated cytosolic form, bound to GDP, and an active membrane-associated state, when bound to GTP. Arf proteins are highly conserved and are present in all eukaryotes examined to date, except for Arf2, which has not been reported to be present in humans. These proteins have been shown to play a number of regulatory roles related not only with membrane traffic, but in lipid metabolism, organelle morphology, and cellular signaling [27,28,29]. Given that Arf1 and Arf3 are the most abundantly expressed Arf isoforms in cells, Class I Arfs have been assumed as the principal actors in vesicle transport. However, in recent years it has been demonstrated that although the different Arf proteins play overlapping and redundant roles in cell transport, specific Arfs display a unique profile of activities to control specific points in the secretory and endocytic pathways. In addition, it has also been observed that in some cases the control of vesicle transport can depend on the cooperation of two particular Arfs [30].

In addition to Arf proteins, the participation of specific Arf GEFs has also been shown to play an essential role in the regulation of the vesicle transport. For instance, the activation of Arf1 can be driven either by GBF1 to promote COPI transport, or by the Brefeldin A-inhibited GEFs 1 and 2 (BIG1 and BIG2) to initiate clathrin transport; thus, it is thought that Arf1 is committed to each transport pathway depending on the interaction with a specific GEF. In order to achieve this regulation, the different GEFs have been shown to be restrained to particular sites where a specific transport is needed; for example, GBF1 is located at sites where COPI exerts its functions, either in the cis-Golgi cisternae, the ERGIC or the LDs [34,35], whereas BIG1 and BIG2 are located at the trans-Golgi network (TGN), where clathrin works. In this way, each transport pathway depends on the precise temporal and spatial interaction of a specific GEF with a combination of small GTPases.

**Figure 3:**
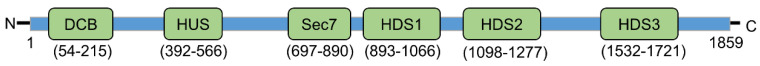
Domain organization of GBF1. The positions of the Sec7, DCB, HUS, and HDS1-3 domains are indicated. The amino acid residues comprised in each domain are shown in parentheses.

GBF1 is the GEF that specifically regulates the COPI vesicle transport. Based on its size, GBF1 belongs to the group of large Arf GEFs, which also contains BIG1 and BIG2. This group of GEFs shares a catalytic Sec7 domain that regulates the activation of Arf proteins through binding to the Arf-GDP form, inducing its conformational change, and promoting the exchange of GDP for GTP that leads to the dissociation of the protein from GBF1 [36,37]. Through this mechanism, GBF1 can activate Arf1, Arf4, and Arf5 [38,39,40]. In addition to its Sec7 domain, GBF1 contains five non-catalytic domains: the N-terminal dimerization and cyclophilin binding (DCB) domain, the homology upstream of Sec7 (HUS) domain, and three C-terminal downstream of Sec7 (HDS1-3) domains [34,41] (Figure 3). The functions of these non-catalytic domains are not well understood, but given that GBF1 cycles between a soluble and a membrane-bound state, similar to Arf proteins, it has been proposed that these domains are important for the specific interaction of GBF1 with cellular membranes [35,42,43,44].

The multidomain structure of GBF1 allows this protein to engage in numerous interactions with other proteins, leading GBF1 to participate in many different cellular processes and even in the process of infection of some bacteria [45,46]. In addition to its canonical role in vesicular transport, GBF1 has also been shown to participate in the function of LDs and mitochondria, as well as in a clathrin-independent endocytosis pathway and the chemotaxis of migrating neutrophils [34]. Many of these functions have been elucidated through the use of the fungal toxin Brefeldin A (BFA) and the small-molecule Golgicide A (GCA), which inhibit the catalytic activity of this nucleotide exchange factor. These inhibitors bind to the protein–protein interface in the complex formed by the Sec7 domain of GBF1 and Arf-GDP, and block the conformational changes of Arf proteins that allow the exchange of GDP for GTP [36]. This inhibition leads to the release of COPI from the membranes with the consequent inhibition of the vesicle transport, and also primes the disassembly of the cisternae of the Golgi apparatus. Moreover, it has also been observed that BFA and GCA induce the swelling of the ER compartment, likely due to the fusion of the cis-Golgi with the ER. Both BFA and GCA share many similitudes in their effects; however, while BFA has been shown to inhibit the activity of GBF1, BIG1, and BIG2 GEFs, GCA does not affect other GEFs as it is a specific inhibitor of GBF1 [15,32,33,47]. These inhibitors have been useful tools for understanding the participation of GBF1 in different cellular functions. Moreover, in recent years, the use of these drugs has unveiled the essential role that GBF1 plays in the replication of several RNA viruses that belong to different families (Table 1), strongly suggesting that GBF1 can exhibit diverse functions that go beyond its role in vesicle transport. In this review, we explore the current knowledge of the role GBF1 plays in the replication of RNA viruses and present an overview of the different viral functions that depend on the activity of this factor.

DENV, dengue virus; TBEV, thick-borne encephalitis virus; YFV, yellow fever virus; KUN, Kunjin virus; CSFV, classic swine fever virus; HCV, hepatitis C virus; CVA21, coxsackievirus A21; CVB3, coxsackievirus B3; CVB4, coxsackievirus B4; EV71, enterovirus 71; HRV2, human rhinovirus 2; HRV14, human rhinovirus 14; CHIKV, chikungunya virus; SINV, Sindbis virus; HEV, hepatitis E virus; MHV, mouse hepatitis coronavirus; SARS-CoV, severe acute respiratory syndrome coronavirus; HCoV-229E, human coronavirus 229E; VSV, vesicular stomatitis virus; HPIV3, human parainfluenza virus 3; IAV, influenza virus; LCMV, lymphocytic choriomeningitis virus.

## 2. Positive-Sense RNA Viruses

### 2.1. Family Flaviviridae

#### 2.1.1. Flavivirus

Flaviviruses are enveloped, positive-sense, single-stranded RNA viruses with a genome of approximately 9.4–13 kb in length. The genome is organized as a long open reading frame (ORF) encoding a polyprotein that is cleaved into three structural (C, prM, and E) and seven nonstructural (NS1, NS2A, NS2B, NS3, NS4A, NS4B, and NS5) proteins. During infection, flaviviruses induce a profound rearrangement of cellular membranes to provide platforms for viral replication. It has been observed that RNA replication occurs in vesicular structures called vesicle packets derived from membranes of the ER [77]. Although the assembly process of flaviviruses is unclear, it has been suggested that during RNA replication, the viral capsid protein (C protein), located on the surface of LDs, recruits the viral genome for formation of the nucleocapsid [78]. The newly formed nucleocapsid then buds into the ER lumen, acquiring during this process the structural glycoproteins E and prM, as well as the membrane envelope. Finally, as the novel viral particle travels through the secretory pathway to be released by exocytosis, the virus experiments a maturation process in which the prM protein is cleaved by the furin protease to yield the mature M protein [77].

In recent years, increasing evidences have shown that GBF1 is an important cellular factor involved in the replication of several flaviviruses, including dengue virus (DENV) [49,78,79,80], yellow fever virus (YFV) [51], tick-borne encephalitis virus (TBEV) [50], and Kunjin virus (KUN) [52]. In the case of DENV, it was shown that BFA and GCA reduce the abundance of viral RNA, and depletion of GBF1 was sufficient to cause this reduction and to inhibit the viral production [49,79]. Also, BFA has been shown to retard the mortality of mice infected with DENV, but not to alter the final mortality outcome [81]. In support of the participation of GBF1 in the DENV lifecycle, it was found that the viral RNA decrease induced by GCA could be reverted by overexpression of either wild-type GBF1 (GBF1-Wt) or a drug resistant GBF1 mutant [47,79]. The mechanism through which GBF1 favors the replication of DENV RNA is unknown; however, the fact that this factor directly interacts with the NS5 protein, the viral RNA-dependent RNA polymerase (RdRp) of DENV, suggests that GBF1 might be directly involved in the RNA replication process through regulation of the activity of NS5 [79]; alternatively, it has also been proposed that GBF1 might modulate the replication of viral RNA by participating in the biogenesis of the viral vesicle packets [79]. In support of this latter idea, it has been reported that BFA can prevent the membrane rearrangement induced by KUN, when the drug is added at early times of infection [52]. Altogether, these findings suggest that GBF1 might engage the nascent replication complexes of flavivirus (vesicle packets) through its interaction with NS5; once in these sites, GBF1 could regulate the formation of these viral organelles through the activation of Arf proteins and the recruitment of cellular effectors that ultimately could lead to the creation of a microenvironment that allows the proper activity of NS5.

In addition to the role of GBF1 in RNA replication, it has been reported that depletion of this factor or its pharmacological inhibition with BFA could also affect the DENV assembly by blocking the accumulation of the C protein into the LDs, which depends on the catalytic activity of GBF1 [48]. Moreover, considering that depletion of other elements involved in COPI transport, such as the COP-β subunit or the simultaneous depletion of Arf1 and Arf4, largely reduce the accumulation of the C protein in LDs, it was suggested that the dynamic delivery of the C protein from ER to LDs is regulated by the COPI complex through the GBF1-mediated activation of Arf1 and Arf4. On the other hand, and similar to DENV, in TBEV-infected cells, the inhibition of GBF1 also showed an alteration in the transport of the structural prM, E, and C proteins [50].

Of interest, the increment of the C protein transport and the inhibition of viral progeny production has also been recapitulated by treating TBEV-infected cells with interferon 1 (INF1), through the induction of the expression of the viperin (virus inhibitory protein, ER-associated, IFN-inducible) protein [50]; viperin also has antiviral effects on other flaviviruses such as DENV [82] and West Nile virus (WNV) [83]. The antiviral activity of viperin seems to be the result of the inhibition of the GBF1 activity, since overexpression of this factor was shown to counteract the effects of viperin on the TBEV infection [50].

On the other hand, it has been shown that the simultaneous depletion of Arf4 and Arf5 decreases the secretion of DENV and YFV particles, without affecting the constitutive secretory pathway [80]. The fact that these Arf proteins can be selectively activated by GBF1, and that the block in the secretion of DENV particles can be replicated by treatment of cells with BFA, suggest that the secretion of DENV particles to the extracellular medium is GBF1-mediated and is dependent on the activation of Arf4 and Arf5. However, this requirement is not shared by all flaviviruses since the replication of WNV is not affected by depletion of these Arfs [80].

#### 2.1.2. Pestivirus

Although many aspects of the pestiviruses replication cycle remain unknown, a recent study described that replication of the classical swine fever virus (CSFV) depends on the activity of GBF1. It was described that both BFA or GCA are able to reduce the viral titers and the viral RNA level, and depletion of GBF1 is sufficient to cause this effect [53]. It seems that GBF1 could facilitate pestiviruses replication by regulating the COPI-mediated transport, since knocking down the expression of Rab2, a regulatory element of the retrograde transport mediated by COPI [84], has been shown to significantly decrease the viral titer and the RNA level of CSFV. Of interest, this transport does not seem to rely on the activation of Arf1, since depletion of this factor did not affect CSFV replication [53]. The replication of bovine viral diarrhea virus (BVDV), the prototype member of the *Pestivirus* genus, was also demonstrated to be strongly inhibited in the presence of BFA [54], suggesting that the dependence on GBF1 could be a feature shared among different pestiviruses.

#### 2.1.3. Hepacivirus

Infection by hepatitis C virus (HCV), the only member of the *Hepacivirus* genus, induces an extensive remodeling of intracellular membranes to produce a membranous web composed of ER membranes, LDs, and double-membrane vesicles containing HCV nonstructural proteins (NS3, NS4A-B, NS5A-B) as well as the HCV RNA [85,86]. Although the biogenesis of this membranous web is not well understood, the NS4B and NS5A proteins appear to play a major role in the induction of membrane rearrangements [87,88]. Once the web is formed, the NS5B protein (RdRp) directs the replication of the viral positive-sense RNA genome. Detailed knowledge about the individual steps of HCV particles assembly is lacking; however, it is generally assumed that nucleocapsid formation and budding are spatially and temporally linked events occurring in an ER-derived compartment [85,86,89]. In this regard, it has been reported that GBF1 facilitates multiple steps of HCV infection, including the replication of viral RNA [54,55]. A decrement in the levels of GBF1 was found to diminish the expression of NS5A, a multifunctional protein that modulates the viral polymerase NS5B; however, most of the effect on virus replication seems to be related to the role of GBF1 in the formation and function of the membranous web where the replication complexes of HCV assemble [54]. GBF1 inhibition neither disrupts the preformed membranous webs of HCV nor blocks the formation of novel membranous web structures, but it rather affects the maturation of these viral organelles, which exhibit a smaller and less organized structure during the inhibition of this factor [54,55]. In this regard, it was reported that the inhibition of GBF1 induces a change in the intracellular localization of NS5A and NS3 (viral protease) from their usual location in the replication complexes to the rims of LDs [90], suggesting that GBF1 could mediate the transport of non-structural viral proteins and perhaps cellular proteins to these sites. In support of this possibility, it has been found that while a BFA-resistant GBF1 mutant could revert the effects of BFA on HCV, an inactive mutant or a truncated form of GBF1 lacking the catalytic Sec7 domain were unable to maintain the replication of HCV in the presence of BFA [54,91]; this suggests that the role of GBF1 depends on its capacity to activate Arf proteins. However, in a contrasting observation, the expression of NS5A was reported to downregulate the activation of Arf1 [90].

Arf1 activation has also been shown to be related with the viral assembly of HCV through modulation of the recruitment of the adipose differentiation-related protein (ADRP) to LDs. ADRP, a member of the perilipin family, is a major protein associated with LDs in various cell types. This protein has been proposed to play a positive role for HCV RNA replication while performing a negative function for HCV assembly [92]. Although the role of this protein in RNA replication is unknown, it has been observed that ADRP shields the recruitment of the HCV core protein into LDs, a step essential for virus morphogenesis [92,93]. In addition, the association of ADRP with LDs has been shown to be dependent on the activation state of Arf1. Altogether, these results suggest that during HCV infection the activation of Arf1 induced by GBF1 promotes the exportation of NS5A and NS3 from the LDs to the sites where replication complexes are located, to favor the replication of the viral RNA and, at the same time, induces the release of ADRP from LDs to favor the morphogenesis of the virus particles. Since BFA treatment has also been shown to inhibit the secretion of HCV viral particles, leading to their progressive intracellular accumulation within the ER [94], it seems that GBF1 is important for both the assembly and exit of the newly formed HCV virions.

Similar to Arf1, the simultaneous depletion of Arf4 and Arf5, in which the activation also depends on GBF1, has been reported to reduce the RNA replication of HCV probably through affecting the morphology of LD but not the secretion pathway [51,91]. Altogether, these results suggest that the role of GBF1 is not restricted to maintain a single type of transport but rather to coordinate different transport pathways that promote the infection of HCV. Moreover, they also indicate that the role of GBF1 in the LD transport, but not in the secretory pathway, is important for HCV infection. In agreement, several works have reported that HCV replication can be completely inhibited at BFA concentrations that do not affect the secretion of proteins [54,55,95].

In addition to the role of GBF1 in the transport of cellular and viral proteins relevant for RNA replication and viral assembly, it has also been observed that this factor can regulate the lipid composition of the membranous web induced by HCV. Inhibition of GBF1 activity and Arf1 activation abolishes the accumulation of phosphatidylinositol 4-phosphate (PI4P) in the membranes of the HCV replication complexes [96], a process that has been shown to promote the replication of the viral RNA. Although it is not clear how GBF1 and Arf1 prompt the accumulation of PI4P, recent findings suggest that these factors might induce the transport to the HCV replication sites of the type III phosphatidylinositol 4-kinases α (PI4KIIIα) and β (PI4KIIIβ) enzymes, which are responsible in the Golgi apparatus for the generation of PI4P. Although the precise role of PI4P in HCV replication remains unknown, it has been proposed that these lipids might be important to generate membranes sites that permit the association of proteins to the replication complex of HCV, but it could also impact the organization and kinetics of the transport pathways modulated by HCV [56,62,97].

In a mechanism apparently different from the regulation of protein traffic, it has been found that GBF1 might also modulate the processing of the HCV polyprotein by regulating the protease activity of the NS3-NS4A complex; this regulation process requires the interaction between the Sec7 domain of GBF1 and the protease domain of NS3. Moreover, the protease activity of the NS3-NS4A complex has been shown to depend on the catalytic activity of GBF1 [98].

### 2.2. Family Picornaviridae

#### Enterovirus

The genus *Enterovirus* (EV) includes a collection of small non-enveloped viruses with a small positive-sense single-stranded RNA genome. These viruses have been classified into 15 serologically distinct groups, comprising 12 groups of enterovirus (EV-A to L), and three groups of rhinovirus (RV) (RV-A to C). The replication cycle of EV starts with the attachment of the virus to specific cell host receptors. After virus internalization, the viral genome is released into the cytoplasm to direct the synthesis of a single polyprotein, which is auto-processed by viral proteases 2A and 3C to produce three structural proteins (VP0, VP1, and VP3) and seven nonstructural proteins (2A–2C and 3A–3D), as well as some stable protein precursors (2BC, 3AB, and 3CD) that can also participate in the replication cycle. As the infection progresses, EV promotes the creation of viral cytoplasmic replication complexes formed by clusters of tightly associated vesicles of heterogeneous size, where the viral RdRp 3D protein directs the replication of the genomic RNA. The novel genomic RNA then is either translated or packaged into preassembled procapsids (composed of VP0, VP1, and VP3). Finally, the newly EV procapsids are secreted into the extracellular medium through a process in which VP0 is cleaved into VP2 and VP4, to form the mature, infectious virus particles.

Although many of the details of the EV replication cycle have been described, the role of host factors in the replication complexes of EV are less characterized. One of the most intriguing features of EV replication is the participation of the cellular secretory pathway in the infectious process. The inhibition of this pathway by BFA and GCA revealed that the RNA replication of many members of EV genus, such as poliovirus, CVB3, coxsackievirus B4 (CVB4, member of EV-B group), coxsackievirus A21 (CVA21, member of EV-C group), enterovirus 71 (EV71, member of EV-A group), echovirus 11 (E11, member of EV-B group), and bovine enterovirus type 2 (BEV2, member of EV-F group) are sensitive to these drugs [51,60,61,64,99,100,101,102,103]. Furthermore, the replication of human rhinovirus 2 (HRV2, member of RV-A group) as well as human rhinovirus 14 (HRV14, member of RV-B group) was also inhibited by BFA and GCA [65]. The reason for this blockage is unknown, although it has been reported that these inhibitors do not alter the formation of the replication complexes of EV, but rather inhibit the proper functioning of these viral organelles, which are unable to support the viral RNA synthesis [58]. Also, it has been shown that the blockage of RNA replication produced by BFA and GCA is related to the activity of GBF1 since the overexpression of GBF1 prevents the effects of these drugs on EV replication [58,61,64,65]. Moreover, GBF1 knockdown confirms that the reduction of this factor reduces the EV RNA replication [58,61,62,63,64,104]. Although the precise role of GBF1 in EV replication is unknown, further analysis of poliovirus, CVB3, and EV71 infection revealed that the N-terminal region of GBF1 interacts with the viral 3A protein; this interaction, in turn, stimulates the association of GBF1 with the membranes where the EV replication complexes are located [58,59,64,105,106,107,108], inducing ultimately a steady increase in the activation of Arf1, Arf3, and Arf5 [64,104,109].

Although it is generally accepted that GBF1 interacts with the 3A protein, the importance of this interaction for the replication of the EV RNA remains to be elucidated. The use of different GBF1 mutants revealed that mutations in this protein that abolish the interaction with 3A reduce the ability of this cellular factor to support EV RNA replication [58,59]. However, it has also been found that an EV mutant strain with a 3A protein incapable of interacting with GBF1 replicates to a similar extent as the wild type virus does [58,105,108], suggesting that the interaction GBF1-3A is dispensable for the EV replication. In this regard, it has also been found that even though GBF1 can be recruited to the replication complexes formed by HRV2 and HRV14, the 3A protein of HRV14 only shows a slight interaction with GBF1, whereas the 3A protein of HRV2 did not interact at all [65,107], indicating that the recruitment of GBF1 to the replication complexes of EV is not dependent on its interaction with 3A. Reconciling these findings is complicated; however, it is possible that mutations in GBF1 that alter its interaction with 3A might also abolish the ability of GBF1 to interact with other cellular factors needed for the proper function of this GEF in the RNA replication of EV, an effect that is not observed when the 3A protein is mutated. Furthermore, taking into account that the expression of 3A can inhibit selectively some steps of the secretory pathway [105,106,110] and that overexpression of GBF1 has been shown to counteract these effects [105], it seems that the interaction of 3A with GBF1 is essential to block the secretory pathway, rather than to participate in the replication of EV. Therefore, it is possible that 3A functions as a competitive inhibitor that binds GBF1 in order to hinder its activity in the secretory pathway.

The relevance of the Arf activation in the replication cycle of EV is also unclear. The depletion of Arf proteins has been shown to reduce the replication of EV71, CVB4, and CVB3 [51,62,64], indicating that these factors are important for EV replication. However, the expression of a truncated mutant composed of the N-terminal DCB and HUS domains of GBF1, but lacking the Sec7 domain, has been shown to be sufficient for the rescue of poliovirus and CVB3 RNA replication in the presence of BFA [59,61]. Thus, it has been proposed that although GBF1 is essential for EV replication, the increment of Arf activation detected during infection of some members of EV genus might be a side effect produced by the increased association of GBF1 with membranes [111]. These results led to propose that the N-terminus of GBF1 might play an essential role in RNA replication by stimulating the recruitment of cellular factors that are essential for the function of the replication complexes of EV; however, the identity of these effectors remains to be elucidated. Recent findings have shown that poliovirus triggers the lipid membrane synthesis of replication complexes by mobilizing the neutral lipids stored in LDs, in an adipose triglyceride lipase (ATGL) and hormone-sensitive lipase (HSL) dependent fashion [112]. These observations, together with described functions of GBF1 in LDs morphology, and its role in prompting the transport of ATGL to LDs [42,113], seem to indicate that GBF1 might support the function of EV replication complexes through regulating the lipid flux from LDs. In this regard, it has been observed that BFA inhibits the increment of lipid synthesis induced by poliovirus infection [99].

In contrast to the strong evidence regarding the relevance of GBF1 for the replication of several enteroviruses, this dependency is not a common feature shared by all members of the *Picornaviridae* family. Encephalomyocarditis virus (EMCV, member of the *Cardiovirus* genus), the foot-and-mouth disease virus (FMDV, member of the *Aphthovirus* genus), as well as the Aichi virus (AiV, member of *Kobuvirus* genus) have been shown to be resistant to the effects of BFA, indicating that GBF1 is not involved in their replication cycle [102,103,114,115]. On the other hand, the dependency on GBF1 does not seem to be limited to the EV genus, since the replication of parechovirus 1 (ParV1, member of the *Parechovirus* genus) is partially sensitive to BFA [102].

### 2.3. Family Togaviridae

#### Alphavirus

This genus includes a large number of mosquito-borne viruses that can infect mammals, birds, fish, reptiles, and amphibians. Alphaviruses consist of enveloped viral particles that contain a single-stranded positive-sense RNA genome that encodes two ORFs. The first ORF directs the synthesis of four non-structural proteins (nsP1-4), whereas the second ORF codes for the structural capsid protein (CP) and two surface envelope glycoproteins (E1 and E2) [116]. Once in the cytoplasm, the viral genome is translated to produce a single polyprotein formed by the four nonstructural proteins. The auto-proteolytic processing of this polyprotein renders the individual nsP1-4 proteins that assemble to form a replicase complex. The replicase complex, together with the genomic RNA and host proteins, then assemble at the plasma membrane to form viral replication compartments called spherules that direct the replication and transcription of the viral RNA. Furthermore, as the infection progresses, the internalization of the spherules from the plasma membrane has been shown to create large cytopathic type 1vacuoles (CPV-1) that can also direct the replication and transcription of viral RNA. Either from spherules or CPV-1, a subgenomic RNA is transcribed from the second ORF to produce a single polyprotein composed of the structural proteins CP, E2, and E1. The auto proteolysis of this polyprotein releases CP in the cytosol and permits the translocation of E1 and E2 into the ER, where they are post translationally modified to yield the final glycoproteins that are then transported by the exocytic pathway to the plasma membrane. Concomitant with these processes, the formation of new icosahedral nucleocapsids occurs in the replication complexes that associate to plasma membrane sites decorated with E2/E1 and then bud to the extracellular medium, producing the mature enveloped particles. [116,117].

Given the involvement of the secretory pathway in the proper processing and transport of E1/E2 to the plasma membrane, this transport has been thought to be important only for virus particle maturation. However, in recent years it was found that the inhibition of protein transport by BFA or GCA could disturb the RNA replication and protein synthesis of Sindbis virus (SINV) [51,67], the prototype member of this genus, as well as of chikungunya virus (CHIKV) [66]. This alteration was found to be associated with a post entry virus replication step that depends on the activity of GBF1, since depletion of this factor was sufficient to reduce the viral RNA replication [66]. Given that alphavirus replication has been shown to induce the formation of membrane vesicles in order to create the viral replication complexes (spherules and CPV-1) that direct the synthesis of the viral RNA, these results led to propose that the function of GBF1 could be to facilitate the efficient replication of the alphavirus RNA genome by promoting the formation of the membrane vesicles required for this process [67]. In support of this model, it has been found that the alphavirus RNA replication depends on the presence of Arf1, Arf3, Arf4, and Arf5 [51,66], as well as on a functional COPI complex [66]. Thus, these results indicate that GBF1 might induce the formation of membrane vesicles through the activation of the COPI transport mediated by different Arf proteins. In agreement with the importance of GBF1 early in infection, it was found that the inhibitory effect of BFA on the replication SINV is lost when this drug is added at late times post infection. Moreover, these data also suggest that the activity of GBF1 is dispensable for the function of the alphavirus replication complexes since BFA does not affect the replication of the viral RNA once these organelles are formed [67,118].

On the other hand, taking into account that Arf4 and Arf5 participate in maintaining the LD morphology, but are not important for protein transport [91], the involvement of these Arf proteins in alphavirus RNA replication might suggest that GBF1 and COPI could also participate in the viral RNA replication through the function of LDs. Although the possible relation between LDs and alphavirus replication has never been tested in mammalian cells, the finding that SINV infection induces the accumulation of LDs in mosquito Aag2 cells as well as in the midgut of *Aedes aegypti* females [119] seems to support the idea that alphavirus might rely on LDs for its replication.

### 2.4. Family Hepeviridae

The family *Hepeviridae* englobes a group of hepatitis E viruses (HEV) that are non-enveloped and contain a positive-sense single-stranded RNA genome that contains two ORFs. The first ORF encodes a non-structural polyprotein (ORF1 protein) that exhibits several functions, such as methyltransferase, protease, RNA helicase, and RdRp, while the second ORF codes for the capsid protein (CP). Due to the low efficiency of these viruses to grow in cell culture, their replication process is poorly understood, although it has been proposed that it might be similar to that of togaviruses [120]. In fact, a recent report revealed that similar to viruses in the *Togaviridae* family, the RNA replication of HEV was sensitive to treatments with BFA and GCA, and that GBF1 plays an essential role in this process although, unlike togaviruses, the inhibition of GBF1 activity by BFA does not affect the formation of the HEV replication complexes.

### 2.5. Family Coronaviridae

#### Coronavirus

The members of this genus are enveloped viruses that contain a large positive-sense, single-stranded RNA genome with several ORFs that direct the synthesis of 16 nonstructural proteins (nsp1 to nsp16), four structural proteins (S, M, E, and N) and, in some viruses, a group of up to eight accessory proteins that are key for the efficient replication of the virus in natural conditions [121]. Once released in the cytoplasm, the viral RNA directs the synthesis of the nonstructural proteins that form the RdRp complex, which transcribes the full-length, negative-sense antigenomic RNA. The coronavirus antigenome is then used for the production of the full-length genomic RNA copies and a set of subgenomic mRNAs of different lengths that direct the synthesis of the structural and accessory proteins. Following their synthesis, the structural proteins S, E, and M are inserted into the ER and then transported along the secretory pathway up to the ERGIC, where the nucleocapsid, formed by the new RNA genome bound to N proteins, buds to form the mature virions. Following assembly, virions are finally released to the extracellular medium by exocytosis. During coronavirus infection, all RNA synthesis processes occur in a replication–transcription complex (RTC), composed of viral and host proteins that are associated to a reticulo-vesicular network (RVN) made of modified intracellular membranes and double-membrane vesicles (DMVs) apparently derived from the ER [121,122].

Recent evidence has indicated that the secretory pathway participates in the formation and function of both RTC and RVN. It has been reported that the inhibition of the secretory pathway by BFA or GCA strongly reduces the RNA replication of different coronavirus strains such as the human coronavirus 229E (HCoV-229E, alphacoronavirus), the mouse hepatitis coronavirus (MHV, betacoronavirus), and the severe acute respiratory syndrome coronavirus (SARS-CoV, betacoronavirus) [51,69,70]. These effects were shown to be related with the specific inhibition of the activity of GBF1 [69,71]. The precise role of GBF1 in coronavirus RNA replication is not clear; however, given that the inhibition of this factor by BFA does not alter the RNA-synthesizing activity of RTCs, but rather decreases the number of these organelles, as well as the number of DMVs in MHV- and SARS-CoV-infected cells, it has been proposed that the GBF1 activity might be required for the formation of the virus-induced RVN [69,70]. Altogether, coronavirus infection seems to redirect the vesicle transport mediated by GBF1 toward the RTCs to maintain the correct morphology of these viral organelles. In support of this proposal, the silencing of Arf proteins or subunits of the COPI complex has shown to induce a similar inhibitory effect on MHV and HCoV-229E replication, similar to that observed with the GBF1 depletion [51,71].

On the other hand, the finding that overexpression of the dominant-negative mutant Arf1-T31N strongly inhibits MHV replication [69], together with the fact that this Arf mutant has been shown to inhibit the ERGIC to ER transport [123], lead to suggest that GBF1 might regulate the maturation of RVN through the retrograde vesicular transport of membrane lipids from the ERGIC to the ER [69].

## 3. Negative-Sense RNA Viruses

### 3.1. Family Rhabdoviridae

#### Vesiculovirus

The *Vesiculovirus* genus comprises a distinct monophyletic group of enveloped bullet-shaped viruses that contain a negative-sense, single-stranded RNA genome that codes for five structural proteins: the nucleocapsid protein (N), the phosphoprotein (P), the matrix protein (M), the glycoprotein (G), and the RdRp (L). After virus entry into the cell, the viral nucleocapsid is released into the cytoplasm. Once free, the virion polymerase complex, composed by the N, P, and L proteins drives the transcription of the viral mRNAs, which then direct the synthesis of the different viral proteins. Once sufficient quantities of the N and P protein have been produced, the virion polymerase complex starts the synthesis of a full-length complementary antigenome RNA of positive sense. The antigenome then serves as a template to synthetize new viral negative-sense genome molecules, which can be used for a second round of transcription or can be assembled into new viral particles. The union of the N protein to the newly genomic RNA compromises these molecules to the formation of helical nucleocapsids that interact with the M protein and are transported to cell membrane sites enriched by the spike G glycoprotein. Budding of the nucleocapsid through these sites leads to the formation of the final enveloped virus [124].

Given that the transport of the G protein to the cell membrane depends on a functional secretory pathway, the vesicular transport was thought to only play a role in the final steps of vesiculovirus replication. However, studies with the vesicular stomatitis virus (VSV), the prototypic member of this genus, revealed that this transport was also important for the synthesis of the viral RNA. It was observed that BFA or GCA treatments induce a substantial reduction in the levels of viral mRNA, and the antigenome RNA of VSV [72,125,126]. The action of these inhibitors was shown to be related to the GBF1 protein [72,125]. Although the exact role of GBF1 in RNA synthesis is unknown, taking into account that the depletion of Arf1 or the overexpression of the dominant-negative mutant Arf1-T31N decrease the abundance of viral RNA products and reduce the VSV gene expression [72], it seems that the role of GBF1 in the process might be related to vesicle transport.

On the other hand, a structure-based computational analysis of possible protein–protein interactions between human proteins and the proteins of Chandipura virus (CHPV), another member of the *Vesiculovirus* genus, which has emerged as a pediatric encephalitic virus in India [127,128], revealed that GBF1 and Arf1 exhibit a high potential for interaction with the G and L proteins of this virus [129]. Although the association of GBF1 and Arf1 with the G protein was rather expected, given the association of the G protein with the secretory pathway, the potential interaction of GBF1 and Arf1 with the polymerase L of CHPV is a surprising finding that might indicate that GBF1 could directly regulate the activity of the L protein during viral RNA replication; the interaction between GBF1 and the L protein was not corroborated by biochemical assays.

On the other hand, considering that the microtubule-dependent transport of viral mRNAs from the VSV inclusions to the cytoplasm has been proved to be essential for their efficient translation [130], it might also be possible that GBF1 promotes the protein expression of vesiculoviruses by facilitating the transport of viral mRNAs out of the inclusions through the activation of Arf1 and the subsequent recruitment of COPI elements. In support of this model, it has been reported that VSV infection can be inhibited by microinjection of an antibody against COP-β that blocks the transport between the ER and Golgi [131].

### 3.2. Family Orthomyxoviridae

#### 3.2.1. Influenza Virus

Influenza viruses are enveloped viruses that contain a segmented negative-sense, single-stranded RNA genome that can be formed by seven or eight RNA segments, depending on the virus strain. The genome of these viruses codes for three proteins that form the viral RdRp (PA, PB1, PB2), a nucleoprotein (NP) that is associated with each genome segment, a hemagglutinin (HA) protein that facilitates virus attachment, and the matrix protein 1 (M1) that acts as an adaptor protein between the viral ribonucleoproteins (RNPs) and the membrane envelope. Moreover, some members also code for a neuraminidase (NA) that contains sialidase activity essential for the releasing of the mature viral particles, and a small integral membrane matrix protein 2 (M2), a proton selective ion channel. In addition to structural proteins, these viruses may also code for two nonstructural proteins (NS1, NS2). After cell entry, the RNPs are released into the cytoplasm; once free, the RNPs are then imported to the cell nucleus, where the viral RdRp synthesizes the mRNAs that direct the synthesis of the viral proteins in the cytoplasm of the cell. During their synthesis, the proteins HA, NA, and M2 are inserted into the ER membrane, and are transported to the sites of viral assembly in the plasma membrane. Concomitant with this process, the NP, PA, PB1, and PB2 proteins are imported into the nucleus and bind the newly synthesized RNA genome segments to form the novel RNPs that are then exported to the cytoplasm and subsequently transported to the sites of viral assembly in the plasma membrane. Finally, the budding of RNPs in the enriched HA, NA, and M2 membrane sites produces the new influenza virus infectious particles [132,133].

Two models have been proposed to explain the RNP traffic to the plasma membrane, and in both cases the interaction of the RNPs with Rab11 is involved [134,135,136]. Recently, it was also reported that, in addition to Rab11, the activity of GBF1 was important for the assembly process of influenza A virus (IAV) [73,74,137]. The block on IAV assembly was found to be related with a decrement on the cell surface of the HA, NA, and M2 viral proteins, as well as with disruption of the transport of RNPs to the plasma membrane [73,74,75,137], indicating that GBF1 facilitates the assembly of IAV by regulating the transport to the plasma membrane of the viral elements that form the IAV virion.

Besides its role in regulating the transport of viral proteins to the plasma membrane, it has been shown that GBF1 interacts specifically with the protein M2 [74]. Although the biological importance of this interaction is unknown, the observation that IAV induces the disruption of the Golgi apparatus [75] makes it possible that through the M2-GBF1 interaction the GBF1 is hijacked from the secretory pathway (as observed for the 3A protein of picornavirus), to promote the transport of HA, NA, M2 proteins, and the RNPs. Moreover, the observation that the inhibition of RNP transport by BFA induces the accumulation of RNPs in tubulated structures in the perinuclear region [137], suggest that M2 might recruit GBF1 from the Golgi apparatus to the tubulate ER network to promote the transport of HA, NA, M2, and RNPs to the plasma membrane. In support of the role of GBF1 to stimulate the transport of these viral elements, it has been found that Arf1 can colocalize with RNPs of IAV [75], suggesting that GBF1 might mediate the traffic of IAV RNPs by maintaining an active COPI transport. In support of this idea is the discovery that RNPs travel to the plasma membrane in a microtubule dependent fashion [134,135], as well as the close relationship between the movement of COPI vesicles and microtubules, which seem to indicate that COPI vesicles might be important to the transport of RNPs. In addition, COPI was also suggested to be related with the internalization process of IAV, since its depletion was shown to reduce this process [73,138]. However, given that these results could not be recapitulated by BFA and GCA treatments, it was presumed that this effect might be due to the long-term inhibition of protein and lipid transport to the plasma membrane [73]. The evaluation of the effects of RNAi depletion of COPI on IAV replication assays that bypass the virus internalization process would help to bring light into this controversial matter.

#### 3.2.2. Other Negative-Sense RNA Viruses

The participation of GBF1 in the replication of negative-sense RNA viruses is not restricted to IAV. The replication of human parainfluenza virus type 3 (HPIV3, member of *Paramyxoviridae* family), as well as that of the lymphocytic choriomeningitis virus (LCMV, the prototypic member of the *Arenaviridae* family) have also shown to be dependent on the GBF1 activity. Depletion of this factor by RNAi or its pharmacological inhibition by BFA or GCA, has shown to significantly reduce the protein synthesis of these viruses [72]. However, whether the role of GBF1 might be related directly to viral protein synthesis or results from defects in the previous steps of the viral cycle, remains to be elucidated. On the other hand, although HPIV3 and LCMV were shown to depend on GBF1, the finding that Arf1 depletion reduces the LCMV protein expression, but does not affect that of HPIV3, suggests that the role of GBF1 in LCMV could be related to the COPI transport, whereas for HPIV3 GBF1 might play an alternative function independent of vesicle transport, although the possibility that HPIV3 could depend on other Arfs cannot be ruled out.

## 4. Double-Stranded RNA Viruses

### Family Reoviridae

#### Rotavirus

Rotaviruses are non-enveloped viruses formed by three concentric layers of proteins that surround a genome composed of 11 segments of double-stranded RNA (dsRNA) that code for six nonstructural (NSP1-6) and six structural (VP1-4, VP6, and VP7) proteins. The inner core of rotavirus is composed by the protein VP2, which encloses the replication machinery formed by the dsRNA segments, the RdRp VP1, and the protein VP3. The intermediate layer, formed by VP6, surrounds the viral cores to form double-layered particles (DLPs). Finally, the addition of the glycoprotein VP7 and the spike protein VP4 onto the DLPs forms the infectious triple-layered particles (TLPs). The replication of rotavirus occurs in cytoplasmic non-membranous electron-dense inclusions termed viroplasms. Replication and packaging of the viral genome into newly synthesized DLPs take place in these inclusions. The novel DLPs then bud into the lumen of ER through membrane sites modified by the presence of NSP4 and VP7, acquiring a transitory lipid envelope bearing the NSP4, VP7, and VP4 proteins. As the new DLPs move into the lumen of the ER, the transient lipid envelope is removed by an unknown process in which NSP4 is eliminated while VP4 and VP7 are assembled to produce the final infectious TLPs [139].

Although many of the steps of rotavirus replication have been elucidated in recent years, the precise mechanism of the final events of rotavirus assembly is not well understood. It is known that NSP4 (a transmembrane ER glycoprotein) plays a crucial role in the last steps of rotavirus assembly. The interaction of NSP4 with VP4 and VP7 [140], together with the binding of NSP4 to the VP6 protein on the surface of DLPs, has been proposed to drive the incorporation of the outer layer proteins to form the final TLPs. However, whether specific cellular pathways or cell proteins are involved in the assembly process remains unknown.

In recent years, the use of genome-wide RNAi screens has shown that the vesicle transport mediated by the COPI/Arf1 machinery is relevant for virus replication, since knocking down the expression of some of the proteins that integrate such machinery significantly impairs the yield of rotavirus progeny [141,142]; similar results are obtained when the vesicle transport is inhibited by BFA treatment of rotavirus-infected cells [76,143]. This inhibition is related to the activity of GBF1, since interfering specifically with this factor by GCA, or depletion of this nucleotide exchange factor by RNAi, significantly reduced the production of new viral infectious particles [76]. In addition, the block in the production of infectious virus was associated with the assembly of the virus surface proteins VP7 and VP4, since both BFA and GCA block the assembly of the viral surface proteins onto DLPs, preventing the production of mature infectious TLPs [76]. The restriction in the assembly of TLPs was shown to be the result of a defective trimerization of VP7, required for its assembly onto DLPs, rather than a block in the budding of DLPs into the ER lumen.

It has been shown that the inhibition of GBF1 by either BFA or GCA, or the depletion of this factor by RNAi, alters the electrophoretic mobility of the viral glycoproteins VP7 and NSP4 [76,143]. Although the altered posttranslational modification of VP7 was initially considered to explain its lack of trimerization, increasing evidence has pointed out that the alteration of NSP4 is rather responsible, since depletion of this protein or alteration of its posttranslational processing with tunicamycin, an inhibitor of N-glycosylation, affects the trimerization of VP7 and restricts the maturation of the transient enveloped viral particles to TLPs. [76,144,145]. In support of this observation, it was recently demonstrated that a recombinant VP7 protein overexpressed in transfected cells formed trimers only when co-expressed with NSP4 [76]. Therefore, it is likely that the altered post-translational modification is suffered by NSP4 when the expression of GBF1 is knocked down, affects its role in facilitating (directly or indirectly) the trimerization of VP7. It cannot be discarded, however, that the altered glycosylation pattern of VP7 in the presence of the pharmacological inhibitors could also have an impact in its trimerization. The altered electrophoretic mobility of VP7 in the presence of drugs or in cells depleted of GBF1 has been shown not to be related to N-glycosylation, O-glycosylation, or phosphorylation [76].

Given that GBF1 functions at the ER-Golgi interface, mediating the retrograde vesicular transport, and that rotaviruses mature in the ER, it seems likely that Golgi-ER transport is the relevant process required for the correct maturation of rotavirus. However, considering that LDs have been proposed to play a significant role in the replication cycle of these viruses, it remains possible that the protein transport between the ER and LDs is also relevant, although, a so far uncharacterized function of GBF1 cannot be discarded.

On the other hand, in support of the requirement of an active GBF1 transport during rotavirus infection, it was found that catalytically inactive mutants of GBF1 failed to support replication of the virus in the presence of BFA, suggesting that GBF1 activity is essential for rotavirus progeny production. However, given that knocking down the expression of Arf1 was found not to affect the production of infectious virus, it is likely that rotavirus replication does not depend on the GBF1/Arf1 transport but might require the activation of other GBF1 substrates, such as Arf4 or Arf5, that could compensate for the reduced level of Arf1. Moreover, it was also found that while the C-terminal domain of GBF1 is dispensable for the viral replication, the first N-terminal amino acids of this factor are essential to support replication of the virus [76]. The requirement of the N terminus of GBF1 for rotavirus replication has also been reported to be essential for the replication of CVB3 and poliovirus (see above). Thus, these results might indicate that GBF1 could bind a rotavirus protein, similar to what has been observed with the picornavirus 3A protein. However, an interaction of GBF1 with a cellular factor might also be important for virus replication. Further experiments have to be carried out to explore in more detail the mechanism through which GBF1 supports the replication cycle of rotavirus replication.

## 5. Concluding Remarks

As intracellular pathogens, the infection of viruses depends on their capacity to replicate successfully within the host cell. The restricted coding potential imposed by the small size of viral genomes had led the viruses to hijack essential systems of cellular functions to facilitate their replication. In this regard, emerging data have shown that many cellular factors are susceptible to be reprogrammed during viral infections to perform novel functions that benefit these pathogens. One of these cellular elements is the guanine nucleotide exchange factor GBF1, which has proved to be essential for the efficient replication of many RNA viruses. It has been shown to be required for different stages of the virus lifecycles, including the formation and maturation of viral replication complexes as well as the coordination of RNA genome replication, virus assembly, and release. The role of GBF1 has been found, principally, to depend on its ability to activate distinct Arf proteins, to regulate different transport pathways that permit the communication between the viral replication organelles and cellular organelles (ER and LDs) involved in virus infection. However, for many viruses, the role of GBF1 in virus replication has also been shown not to depend on the catalytic activity of the protein. Although it is not well understood how GBF1 could exert this non-catalytic activity, the multidomain structure of the protein seems to play an essential role by permitting GBF1 to engage in the interaction with many cellular and viral proteins that seems to regulate, directly or indirectly, viral replication. The relevance of GBF1 for the replication of several RNA viruses identifies this factor as a potential candidate for antiviral therapies to control a broad-spectrum of viral infections. However, given the essential role of GBF1 in the secretory pathway, and in other so far ill-characterized functions of the cell, the development of safe and effective antiviral therapies that could help to deal with viral infections, without harming the host cells, represents a challenging mission.

In conclusion, the data discussed in this review bring to light a new diverse range of functions of GBF1 that might explain how this factor serves for so many processes in different compartments of the cell. Future studies aimed to clarify the mechanisms underlying the multiple functions associated with this interesting molecule would help to advance our understanding of virus and cell biology.

## Figures and Tables

**Table 1:** Role of GBF1 in the replication of RNA viruses.

Type of Genome	Family	Virus	Viral Replication Step that Requires the GBF1 Activity	Reference
**Non-segmented** **(+)ssRNA**	*Flaviviridae*	DENV	RNA replication Virus assembly	[48,49]
		TBEV	Virus assembly	[50]
		YFV	Virus secretion	[51]
		KUN	Formation of viral replication complexes	[52]
		CSFV	RNA replication	[53]
		HCV	RNA replication Protein expression Virus secretion	[54,55,56]
	*Picornaviridae*	Poliovirus	RNA replication	[57,58,59]
		CVA21	RNA replication	[60]
		CVB3	RNA replication	[60,61,62]
		CVB4	RNA replication	[51]
		EV71	RNA replication Protein expression	[60,63,64]
		HRV2	RNA replication	[65]
		HRV14	RNA replication	[65]
	*Togaviridae*	CHIKV	RNA replication Early protein expression	[66]
		SINV	RNA replication Protein expression	[51,67]
	*Hepeviridae*	HEV	RNA replication	[68]
	*Coronaviridae*	MHV	RNA replication	[69]
		SARS-CoV	RNA replication RNA transcription	[70,71]
		HCoV-229E	RNA replication	[51]
**Non-segmented** **(-)ssRNA**	*Rhabdoviridae*	VSV	RNA replication RNA transcription Protein expression	[72]
	*Paramyxoviridae*	HPIV3	Protein expression	[72]
**Segmented** **(-)ssRNA**	*Orthomyxoviridae*	IAV	Virus assembly	[73,74,75]
	*Arenaviridae*	LCMV	Protein expression	[72]
**Segmented dsRNA**	*Reoviridae*	Rotavirus	Virus assembly	[76]
